# Bi-directional elucidation of *Lactiplantibacillus plantarum* (RTA 8) intervention on the pathophysiology of gut-brain axis during *Salmonella* brain infection

**DOI:** 10.1186/s13099-022-00484-2

**Published:** 2022-03-02

**Authors:** Amrita Kaur, Indu Pal Kaur, Kanwaljit Chopra, Praveen Rishi

**Affiliations:** 1 Department of Microbiology, Basic Medical Sciences Block I, Panjab University, South Campus, Sector 25, Chandigarh, 160014 India; 2 University Institute of Pharmaceutical Sciences, Panjab University, Sector 14, Chandigarh, 160014 India

**Keywords:** *Salmonella*, Neurological complications, Gut-brain axis, Probiotics

## Abstract

**Background:**

There have been reports of patients suffering from typhoid fever, particularly those involving infants and immunocompromised patients, which at times present with *Salmonella* induced brain infection. Although rare, it has frequently been associated with adverse neurological complications and increased mortality. In this context, the gut-brain axis, involving two-way communication between the gut and the brain, holds immense significance as various gut ailments have been associated with psychiatric complications. In turn, several neurodegenerative diseases have been associated with an altered gut microbiota profile. Given the paucity of effective antimicrobials and increasing incidence of multi-drug resistance in pathogens, alternate treatment therapies such as probiotics have gained significant attention in the recent past.

**Results:**

In the current study, prophylactic effect of *Lactiplantibacillus plantarum* (RTA 8) in preventing neurological complications occurring due to *Salmonella* brain infection was evaluated in a murine model. Along with a significant reduction in bacterial burden and improved histoarchitecture, *L. plantarum* (RTA 8) administration resulted in amelioration in the level of neurotransmitters such as serotonin, norepinephrine and dopamine in the gut as well as in the brain tissue. Simultaneously, increased gene expression of physiologically essential molecules such as mucin (MUC1 and MUC3) and brain-derived neurotrophic factor (BDNF) was also observed in this group.

**Conclusion:**

Present study highlights the potential benefits of a probiotic supplemented diet in improving various aspects of host health due to their multi-targeted approach, thereby resulting in multi-faceted gains.

## Background

Neurological complications occurring due to *Salmonella* infection of the brain remain a matter of serious concern [1]. Such infections are associated with frequent relapse episodes, neurological abnormalities along with severe side effects such as auditory and visual impairments, mental retardation and poor prognosis leading to high mortality rates [2]. Previous reports in mouse models have also elaborated on the ineffectiveness of antibiotics in completely curing infections of such kind [3]. Simultaneously, increased incidence and rise in cases of drug resistant *Salmonella* has further limited the treatment arsenal for this dangerous pathogen [4]. Interestingly, recent evidences have indicated that inflammatory diseases/conditions of the gut are linked to psychiatric and behavioural abnormalities [5]. Additionally, various neurodegenerative diseases have been associated with an altered and specific microbiota profile [6]. In the prevailing scenario, tackling and treating infections by targeting multi-dimensional aspects of health and disease has led to increasing interest in the field of neurogastroenterology which interlinks diverse aspects of neuroscience, gastroenterology, immunology, behaviour science, microbiology, pharmacology and other related subjects to devise strategies that can improve the host health status at a holistic level [7]. Herein, manipulation of gut-brain axis has proven to be of enhanced significance, given its direct implications at the gut as well as the brain [8]. The brain to gut signalling is evidenced by the findings that different kinds of psychological stressors modulate the composition as well as the biomass of the gut microbiota. The CNS has been reported to impact other bodily as well as gut functions, including the gut microbiota, via ‘the emotional motor system (EMS)’ consisting of hypothalamus–pituitary–adrenal (HPA) axis, parallelly working branches of the sympathetic as well as parasympathetic autonomic nervous system (ANS), and other pathways mediating discomfort and pain [9]. The ANS finds direct role in gut functions such as mucus, bicarbonate and acid secretion along with intestinal motility, immune response and permeability, coupled with secretion of neuroactive signalling molecules such as catecholamines, cytokines, serotonin, etc., thereby impacting the microbiota [10]. Simultaneously, microbes have been known to influence almost all major pathways associated with the gut-brain axis. These include the neural pathway by modulating/producing/regulating the level of neurotransmitters [11]; endocrine pathways and its associated moieties such as neuroendocrine cells, neuroactive substances and neuropeptides; host immune system as well as functioning and maturation of most components of innate and adaptive immunity. All these molecules have been reported to influence key aspects of brain and behaviour including neurodegeneration, apoptosis and neurogenesis, which, coupled with inflammation, further highlight their importance in maintaining host well-being [12, 13].

Therefore, given the crucial role of gut microbiota in this bi-directional communication, use of probiotics as a bio-compatible treatment strategy has particularly captured the interest of the scientific community. Probiotics have been reported to influence all the key pathways that can be influenced by the host microbiota thereby presenting as a highly promising candidate for targeting gut-brain axis disorders [13]. Additionally, the capability of these bacteria to function as psychobiotics by improving the psychological status along with their impact on the host pathology and physiology has opened diverse areas of targeted therapy which might prove useful in a variety of related manifestations [14]. In our previous study, administration of *Lactiplantibacillus plantarum* (RTA 8) was found to be useful in ameliorating *Salmonella* induced brain infection in mouse model [15]. To the best of our awareness, this is the first study elaborating on the prophylactic effect of *L. plantarum* (RTA 8) in modulating the amount of neurotransmitters and other neuroactive molecules such as BDNF along with mucin genes, at the gut-brain axis, thereby preventing *Salmonella* induced neurological manifestations.

## Results

### Efficacy of *L. plantarum* (RTA 8) at systemic level

**Fig. 1:**
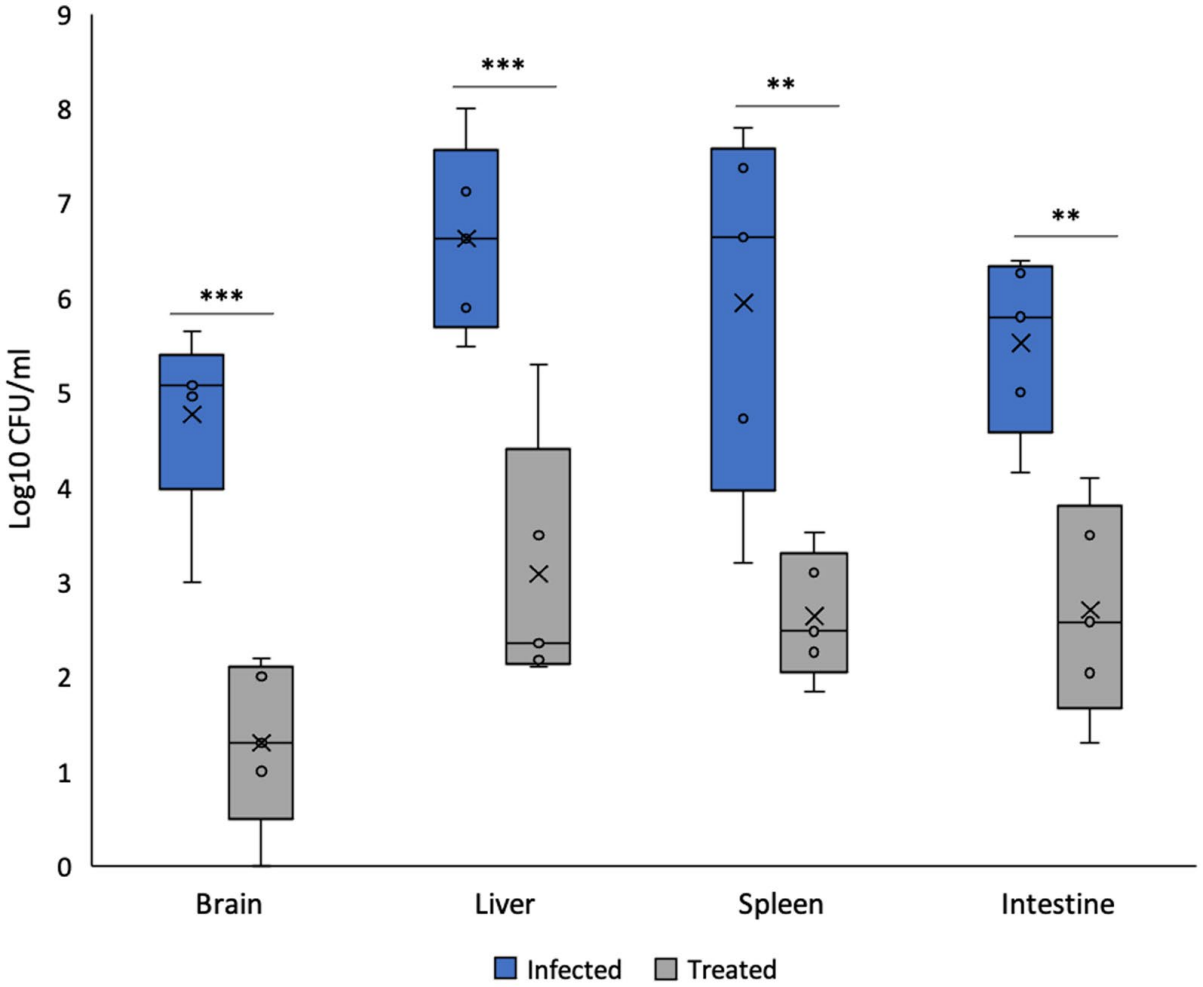
*L. plantarum* (RTA 8) administration 7 days prior to infection significantly reduced *Salmonella* count in all the vital organs. Data has been represented in the form of box and whiskers plot with whiskers representing the maximum and minimum values, boxes representing the 25th (1st quartile) and 75th percentile (3rd quartile), line representing the median, dots representing individual values and ‘x’ representing the mean value of dataset. **p < 0.01 and ***p < 0.001 versus infected by analysis of variance (ANOVA)

Significant bacterial burden in vital organs of mice in the infected group indicated systemic spread of the pathogen. *L. plantarum* (RTA 8) administration 7 days prior to infection significantly reduced *Salmonella* bio-burden in all organs. A 2.9 (*p* < 0.001) and 2.85 (*p* < 0.01) fold reduction was observed in liver and spleen tissues samples of the treatment group, respectively, in comparison to that of the infected group thereby indicating efficacy of the treatment (Fig. 1).

### Efficacy of *L. plantarum* (RTA 8) at gut-brain axis

#### Tissue bio-burden

Dissemination of *Salmonella* from the gut to the brain was confirmed as significant bacterial burden was observed in the brain tissue. Simultaneously, a significant reduction in bacterial burden in the intestine as well as the brain tissue samples of mice in *L. plantarum* (RTA 8) treated group was observed. A 2.84 fold (*p* < 0.01) reduction in *Salmonella* bio-burden was observed in intestinal sections of the treatment group as compared to that in the infected group. Similarly, administration of *L. plantarum* (RTA 8) resulted in a significant decrease in bacterial counts (6.06 fold reduction, *p* < 0.001) to 0.77 log_10_ CFU/ml in the brain tissue samples relative to the infected group, demonstrating the effectiveness of *L. plantarum* (RTA 8) in preventing *Salmonella* brain infection (Fig. 1).

#### Level of dopamine

Infection with *S*. Typhimurium SL1344 significantly reduced the level of dopamine in murine gut tissue samples by 80.9% (*p* < 0.01) in comparison to the control. *L. plantarum* (RTA 8) administered group demonstrated an increase in the level of dopamine, however, the levels were not found to be significantly different from that of the infected group.

**Fig. 2:**
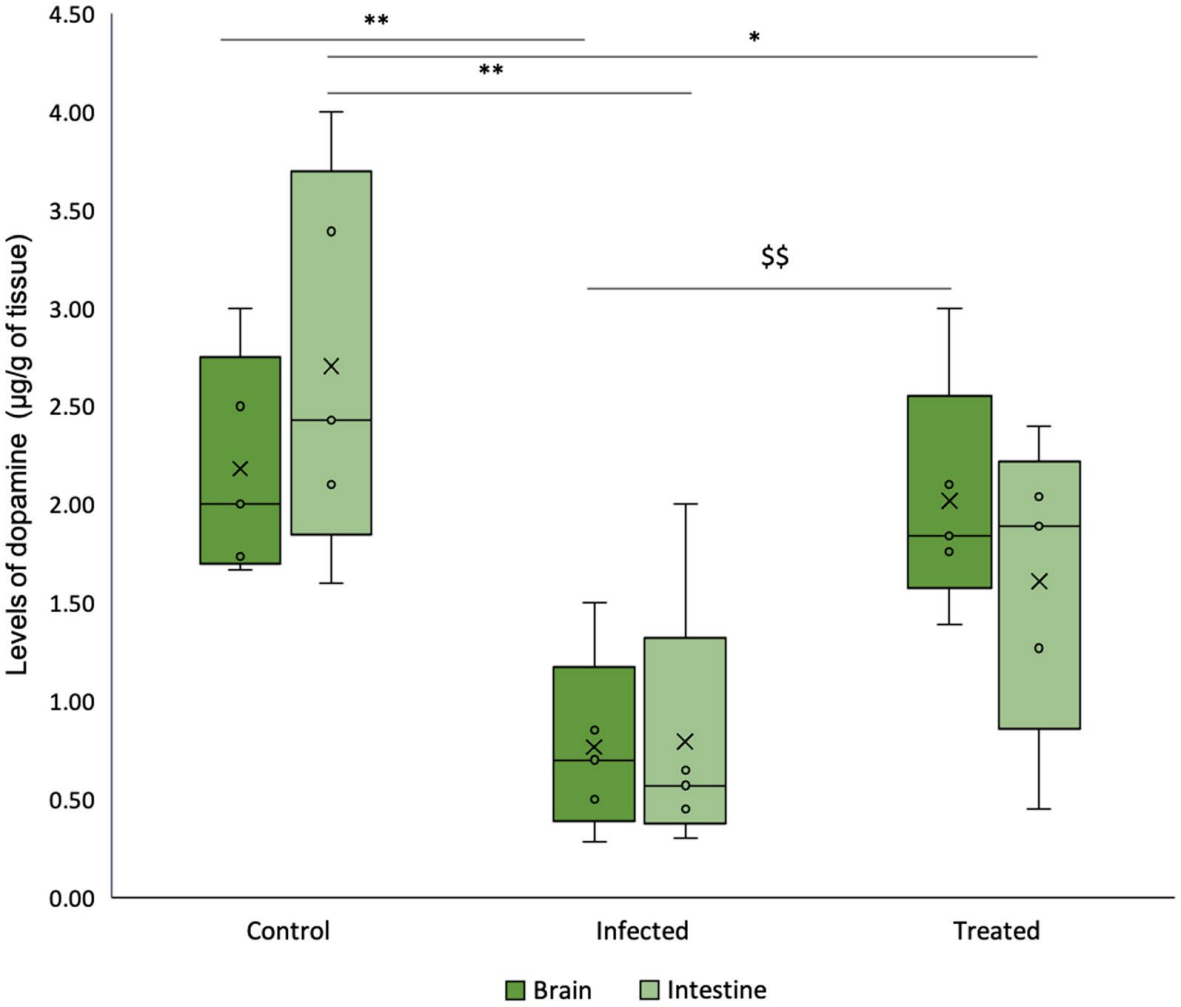
Dopamine levels in the murine brain and intestinal tissue samples. Dopamine levels was observed to be significantly decreased in the infected group and normal levels were found to be restored in murine brain after administration of *L. plantarum* (RTA 8). Data has been represented in the form of box and whiskers plot with whiskers representing the maximum and minimum values, boxes representing the 25th (1st quartile) and 75th percentile (3rd quartile), line representing the median, dots representing individual values and ‘x’ representing the mean value of dataset. **p < 0.01, *p < 0.05 in comparison to Control. ^$$^*p* < 0.01 in comparison to Infected

In comparison to the control group, *Salmonella* infected mice demonstrated a significant reduction of 64.1% (*p* < 0.01) in dopamine levels in the brain tissues. Treatment with *L. plantarum* (RTA 8) significantly increased the levels of dopamine in the brain tissues in comparison to the infected group (*p* < 0.01). Dopamine levels in the treatment group were found to be restored to the level observed in the control group (Fig. 2).

#### Level of norepinephrine

Intestinal samples of mice infected with *Salmonella* demonstrated a decrease of 46.1% in norepinephrine levels in comparison to the control group, however it was found to be insignificant. In comparison to the infected group, mice in the *L. plantarum* (RTA 8) administered group demonstrated a relative increase in norepinephrine levels but the results were not found to be significant.

**Fig. 3:**
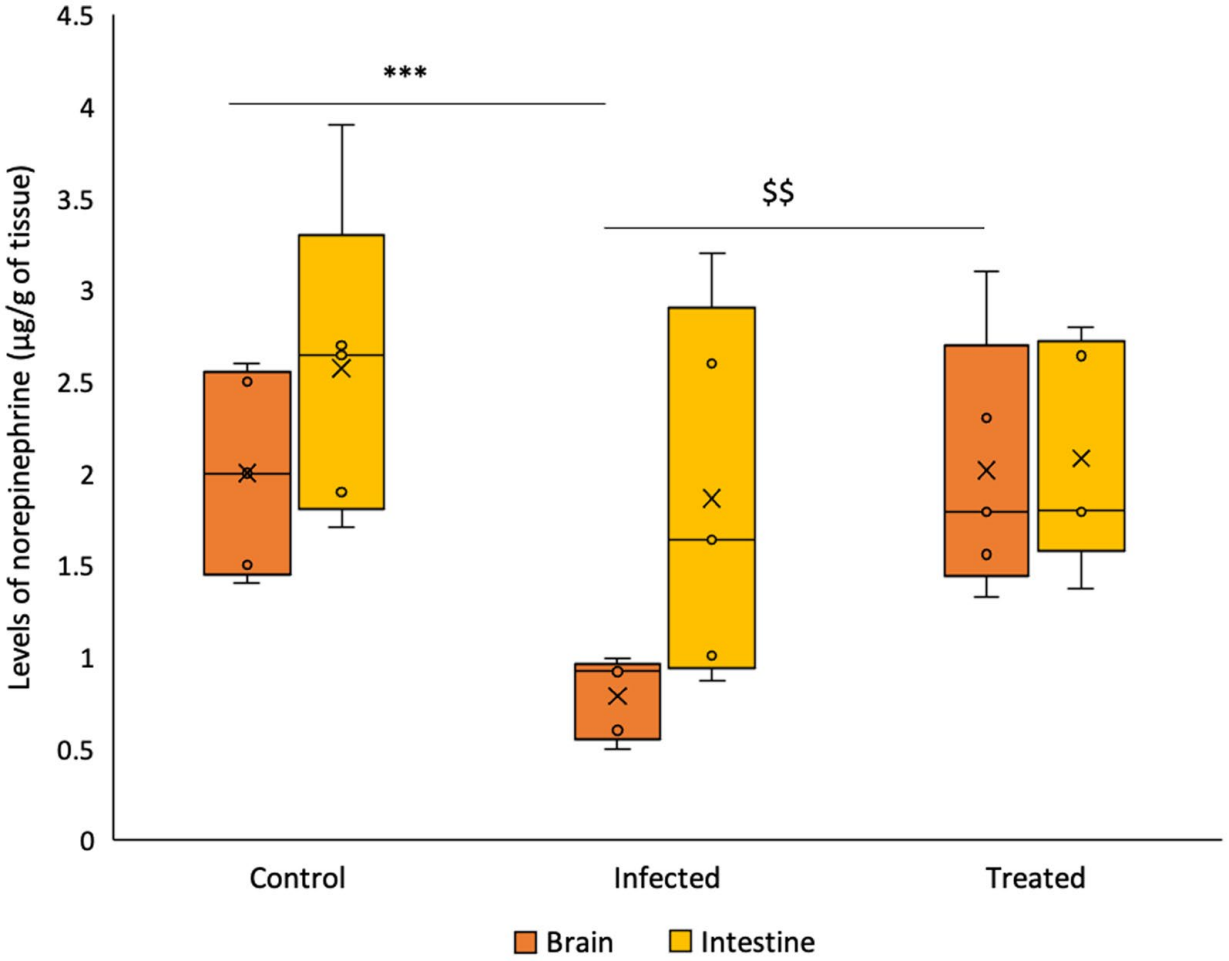
Norepinephrine levels in the murine intestinal and brain tissue samples. Decreased level of dopamine was observed in the *Salmonella* infected group with significantly reduced levels in the brain. *L. plantarum* (RTA 8) administration was found to increase intestinal norepinephrine levels but significant increase was found in the brain tissue samples. Data has been represented in the form of box and whiskers plot with whiskers representing the maximum and minimum values, boxes representing the 25th (1st quartile) and 75th percentile (3rd quartile), line representing the median, dots representing individual values and ‘x’ representing the mean value of dataset.***p < 0.001 in comparison to Control; ^$$^*p* < 0.01 in comparison to Infected

In contrast to results obtained in the intestinal tissue samples, *Salmonella* infected group demonstrated a very significant reduction of 34.7% in the level of norepinephrine in brain tissues samples (*p* < 0.001) as compared to the control group. Treatment with *L. plantarum* (RTA 8) resulted in a significant increase (*p* < 0.01) in the hormone levels in comparison to the infected tissue (Fig. 3).

#### Level of serotonin

*L. plantarum* (RTA 8) administered group demonstrated a significant 34% increase (*p* < 0.05) in the level of serotonin in the gut of mice in comparison to the control group. An 18.4% reduction in the level of serotonin was observed in the intestine tissue samples of mice in the infected group. As compared to the infected group, a significant increase was observed in the treatment group (*p* < 0.01).

**Fig. 4:**
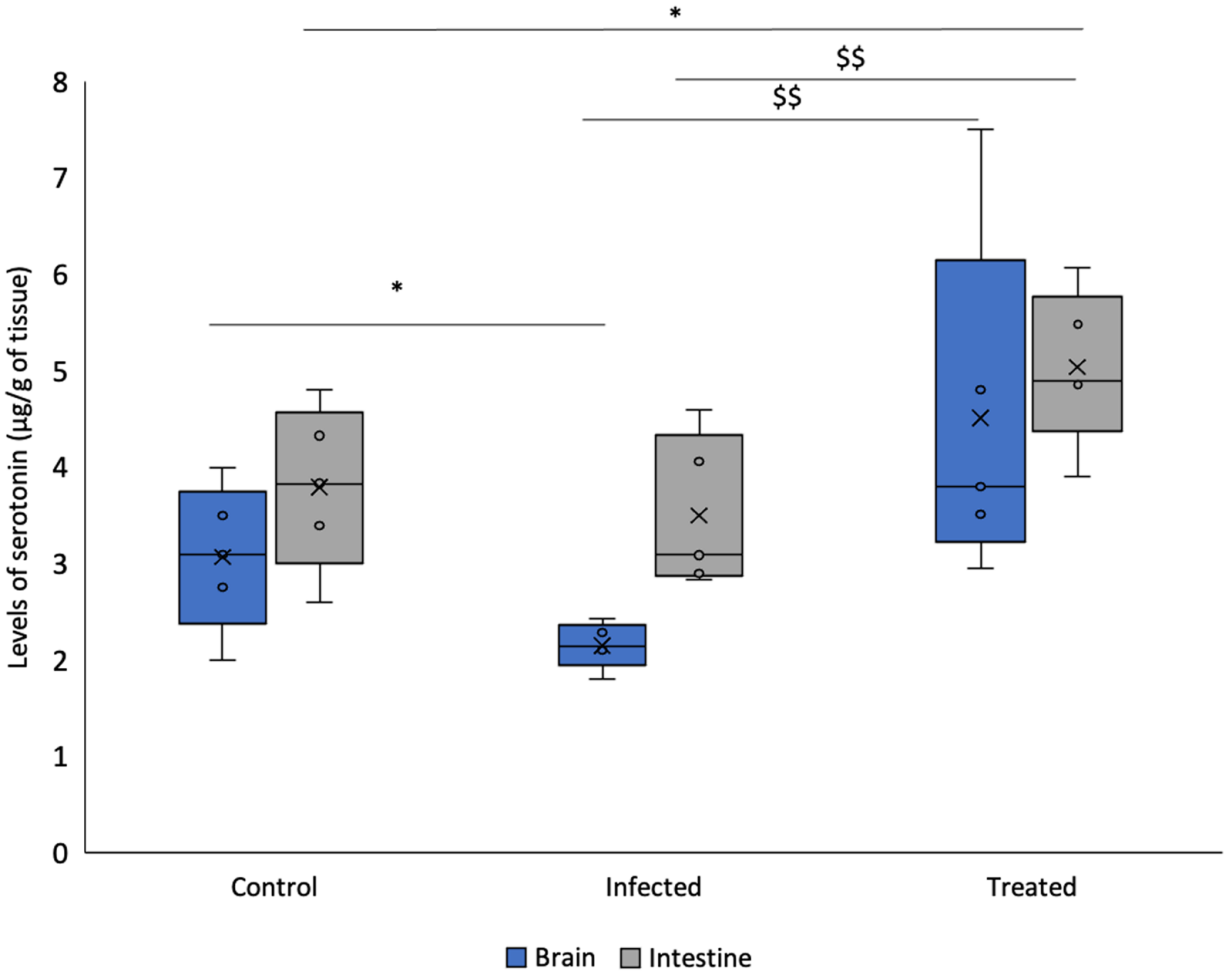
Serotonin levels in the murine intestinal and brain tissue samples. Significantly decreased levels of serotonin were observed in brain tissues of mice infected with *Salmonella*. *L. plantarum* (RTA 8) treated group demonstrated a significant increase in the level of serotonin in the brain as well as the intestinal tissue samples. Data has been represented in the form of box and whiskers plot with whiskers representing the maximum and minimum values, boxes representing the 25th (1st quartile) and 75th percentile (3rd quartile), line representing the median, dots representing individual values and ‘x’ representing the mean value of dataset. *p < 0.05 in comparison to Control; ^$$^*p* < 0.01 in comparison to Infected

In the brain tissue sections, mice in the *Salmonella* infected group demonstrated a significant decrease of 21.9% in the level of serotonin as compared to the control group (*p* < 0.05). Simultaneously, *L. plantarum* (RTA 8) administered group demonstrated a significant increase in the level of serotonin in comparison to the infected group (*p* < 0.01) (Fig. 4).

#### Expression of MUC 1 gene

**Fig. 5:**
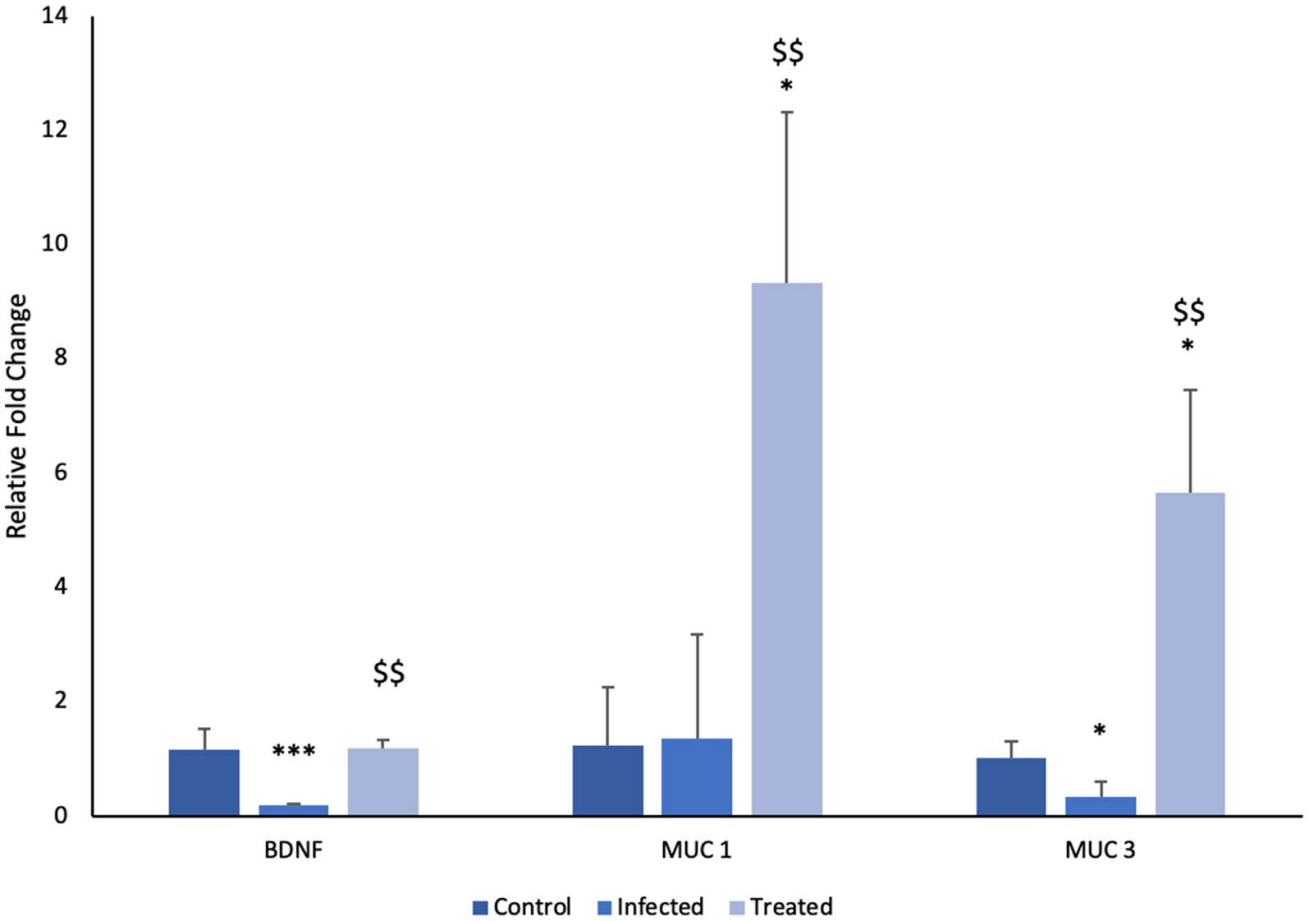
Relative fold change in expression of BDNF, MUC1 and MUC3 genes. Fold expression was determined after normalization to GAPDH for each sample. ***p < 0.001, *p < 0.05 in comparison to control; ^$$^*p* < 0.01 in comparison to infected

Expression of MUC 1 gene was found to be significantly upregulated in the group administered with *L. plantarum* (RTA 8). A 7.6 fold significant increase (*p* < 0.05) was observed in this group as compared to the control group. Additionally, a 6.7 fold increase (*p* < 0.01) was observed in the treatment group as compared to the infected group. No significant difference in gene expression was observed between the control and the infected group (Fig. 5).

#### Expression of MUC 3 gene

Expression of MUC 3 gene in the *Salmonella* infected group was found to be downregulated significantly (3.1 fold, *p* < 0.05). Simultaneously, *L. plantarum* (RTA 8) treated group demonstrated a significant upregulation of MUC 3 gene expression (5.5 fold *p* < 0.05) as compared to the control group. A significant 17.4 fold increase in the expression of MUC 3 gene was observed in the *L. plantarum* (RTA 8) administered group in comparison to the infected group (Fig. 5).

#### Expression of BDNF gene

Quantitative RT-PCR studies revealed significant downregulation of BDNF gene expression in brain tissues of *Salmonella* infected mice. A 6.3 fold reduction (*p* < 0.001) in expression was observed in the infected group in comparison to the control group. *L. plantarum* (RTA 8) administration resulted in a significant 6.5 fold increase in BDNF expression as compared to the infected group (*p* < 0.011) (Fig. 5).

#### Histopathological studies

**Fig. 6:**
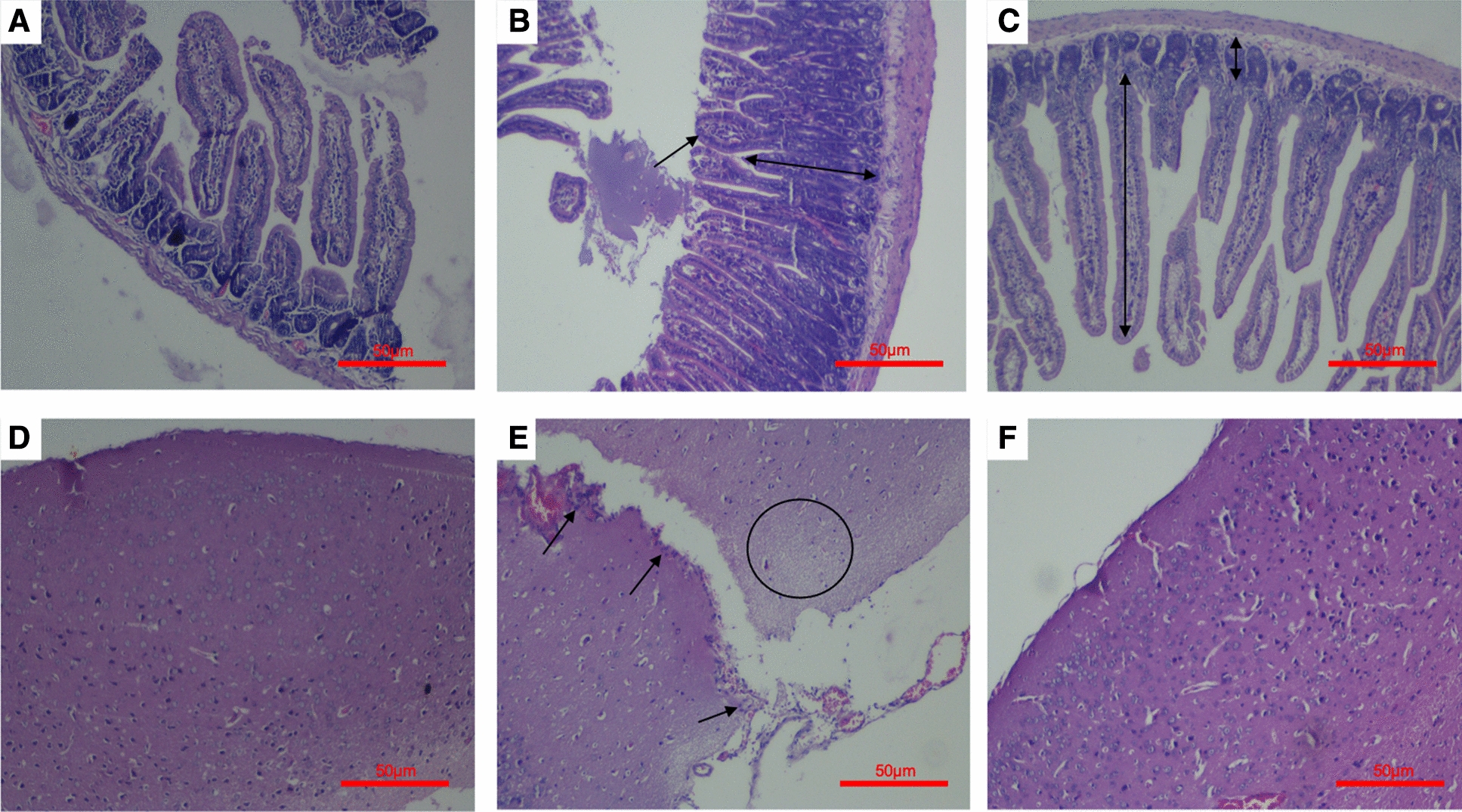
Histological evaluation of brain and intestinal tissue sections. **A** (X40, scale bar 50 µm) H&E staining of control intestinal tissue depicted normal intensity of goblet cells and lymphocytes in the lamina propria depicting normal tissue morphology. **B** (X40, scale bar 50 µm) Photomicrographs of *Salmonella* infected intestinal tissue depicted ileal damage inflicted by *Salmonella* with heavy influx of inflammatory cells along with crypt elongation (highlighted by arrows). **C** (X40, scale bar 50 µm) *L. plantarum* (RTA 8) treated group revealed restoration of normal intestinal tissue morphology as revealed by restoration of villi to crypt ratio and normal density of lymphocytes (highlighted by arrows). **D** (X40, scale bar 50 µm) Photomicrographs of cortical mice brain region in the control group depicted normal histology revealing presence of triangular neurons and glial cells. **E** (X40, scale bar 50 µm) Photomicrographs of brain tissue sections in the *Salmonella* infected group revealed heavy influx of inflammatory cells along the surface and meninges depicting meningitis (highlighted by arrows) along with brain infection characterized by oedema (highlighted by marking a circle). **F** (X40, scale bar 50 µm) Brain tissue sections of mice administered with *L. plantarum* (RTA-8) administered group depicted slight oedema with otherwise normal histoarchitecture

Histopathological analysis of intestinal tissue sections of the control group depicted normal tissue architecture with normal distribution of surface enterocytes, goblet cells and muscularis mucosa. Inflammatory cells were absent and the crypts and villi demonstrated normal histoarchitecture (Fig. 6A). However, infected mice revealed severely damaged villi with focal ulcerations and inflammation. Crypt hyperplasia was also observed. The villi were found to be stunted and the crypts were found to be enlarged and elongated (Fig. 6B). In contrast to this, *L. plantarum* (RTA 8) administered mice demonstrated normal tissue morphology similar to that observed in the control tissue samples. The normal villi to crypt ratio of 5:1 was restored, thereby elaborating on the efficacy of *L. plantarum* (RTA 8) in preventing *Salmonella* infection (Fig. 6C).

Brain tissue samples of mice in the control group revealed normal tissue morphology with the presence of triangular neurons and microglial cells with oligodendrocytes; *sans* inflammation (Fig. 6D). However, oral infection with *Salmonella* led to brain infection and meningitis which was confirmed histologically by increased accumulation of inflammatory cells in the meninges of the brain. Simultaneously, marked oedema was observed in the cortical tissue (Fig. 6E). On the other hand, mice administered with *L. plantarum* (RTA 8) 7 days prior to infection, prevented meningitis as the meninges was found to be clear of signs of inflammation. The brain tissue in this group showed normal tissue histoarchitecture with only very mild oedema (Fig. 6F).

## Discussion

In contrast to the previously conducted independent study [15], herein, exclusively the prophylactic potential of *L. plantarum* (RTA 8) in combating *Salmonella* induced brain infection was evaluated. It was inferred that decreasing the duration of *L. plantarum* (RTA 8) administration from 14 to only 7 days prior to infection, did not affect its efficacy in preventing brain infection. The same was evidenced by a similar reduction in bacterial burden and amelioration in tissue histology, as observed in the previous study. Other studies employing probiotic strains for similar duration have also reported similar fold reduction in *Salmonella* bio-burden [16]. Infection with *S*. Typhimurium has been reported to cause destruction of the ileum, similar to that observed in our study, thereby resulting in increased translocation of bacteria to other organs and imbalance in gut microbial population [17]. Reduction in luminal pH due to production of short chain fatty acids by *L. plantarum* (RTA 8) along with production of IgA and other potent antimicrobial substances might have resulted in clearance of *Salmonella* [18, 19]. Additionally, the abilities of probiotics in occupying receptor binding sites at the epithelial surface, strengthening the intestinal microstructure and increasing the villus length, might have further blocked pathogen adherence [15, 20, 21]. All these factors might have conferred colonization resistance to the host against such deadly pathogens, thereby preventing their translocation to other organs such as the brain. Simultaneously, direct transmission of information from gut to brain through the vagus nerve might have resulted in activation of Fos immune reactive cells in the hypothalamic region of the brain which might have also contributed to effective bacterial clearance from the brain [22]. In consonance with our findings, various studies have highlighted the ability of probiotics in reversing pathogen or microbial component such as lipopolysaccharide (LPS) mediated neurodegeneration by using interventions targeting the gut microbiota [13, 23].

Neurotransmitters constitute a vital part of the gut-brain communication. As observed in the present study, decreased level of serotonin, more significantly so in the brain tissue samples of the *Salmonella* infected group, have been associated with increased pathogenesis and decreased host survival in case of infections caused by pathogens such as *Citrobacter rodentium* and enterohaemorrhagic *Escherichia coli* (EHEC); presence of serotonin has been shown to decrease the expression of LEE virulence genes located within the locus of enterocyte effacement (LEE) pathogenicity island (PI) via the blocking of CpxA, a membrane-bound histidine sensor kinases (HKs) present in bacteria thereby reducing its pathogenicity [24]. Significantly increased levels of serotonin in the *L. plantarum* (RTA 8) group might have resulted in reduced pathogenicity of *Salmonella*, thereby preventing serious consequences. Other studies have reported the ability of probiotic strains such as *E. coli* Nissle 1917 in increasing the bioavailability of serotonin in ileal tissue [25]. The same has been postulated to be a result of SCFA (short chain fatty acids) produced by these gut bacteria acting directly on the enterochromaffin cells which harbour the enzyme tryptophan hydroxylase, resulting in serotonin regulation [26]. Similar to our observations, other studies have also reported increased levels of serotonin in the brain tissues of animals receiving probiotics such as *Akkermansia muciniphila* and *Bifidobacterium breve* CCFM1025 [27, 28]. These effects were found to be mediated by the upregulations of TPH1 and TPH2 genes involved in serotonin biosynthesis in the intestine and the brain, respectively.

The significantly reduced levels of dopamine in the brain and intestinal tissue samples of mice in the *Salmonella* infected group could be attributed to the ability of *Salmonella* and its major cell wall component, LPS, in activation of microglial cells. This might have resulted in a neurotoxic environment thereby increasing the susceptibility of dopaminergic neurons in the substantia nigra and striatum to neuroinflammation induced damage, leading to reduction in its synthesis as well as effective concentration [29]. The normalization of levels of dopamine in the brain tissue samples of mice in *L. plantarum* (RTA 8) treated group could be ascribed to the ability of gut microbiota and probiotics in altering the levels of neurotransmitters in the brain, directly by influencing the ENS and interacting with the glial cells of the gut [12, 30]. Administration of cocktail of probiotics or probiotic strain coupled with a prebiotic have been demonstrated to prevent neurodegeneration while simultaneously increasing the cortical levels of dopamine, as well as preventing degeneration of dopaminergic neurons in the substantia nigra pars compacta [31]. Simultaneously, increased production of butyrate and BDNF/GNDF after probiotic administration, as observed in our study, has also been implicated in raised dopamine levels [32]. In another study, administration of *Lacticaseibacillus zeae* LB1 prevented *Salmonella* infection in *Caenorhabditis elegans* via modulation of both serotonin and dopamine, confirming their role in preventing serious manifestations [33].

Catecholamines have been reported to induce the growth of enteric pathogens by acting as quorum sensing molecules. However, significantly decreased levels of norepinephrine in the brain tissue samples of *Salmonella* infected mice were observed which could be attributed to the role of LPS in reducing the levels of noradrenaline by affecting the brain physiology [34]. Brain infection with *Toxoplasma gondii* has also been associated with decreased level of norepinephrine which might have resulted from changes in the metabolic enzyme tyrosine hydroxylase, that converts tyrosine to catecholamines in noradrenergic neurons [35]. Further, administration of *L. plantarum* (RTA 8) was observed to normalize the level of norepinephrine and could be explained by the previously reported ability of probiotics as well as gut microbiota in modulating the level of catecholamines [36]. Interestingly, host norepinephrine/epinephrine levels have been implicated in downregulation of two important virulence gene of *Salmonella* Typhimurium SL1344 i.e., *virK* and *mig14*, and simultaneously increasing its sensitivity to host antimicrobial peptide LL-37 (which is a part of the innate defence system) suggesting its role in decreasing virulence of the organism [37]. Probiotic strains such as *Lacticaseibacillus rhamnosus* GG and *E. coli* CFR 16 have been reported to increase levels of norepinephrine and restore level of other neurotransmitters via vagus nerve mediated pathways or by directly affecting their receptors [38, 39].

Treatment with *L. plantarum* (RTA 8) resulted in a significant increase in the expression of MUC1 as well as MUC3 mucin genes in the treatment group. Other studies have also reported an increase in mucin production after oral administration of probiotic strains such as VSL#3 or on probiotic exposure to colonic loops and cell lines [40, 41]. The expression of these genes was evaluated given the role of MUC1 in maintaining mucosal barrier during infections such as those caused by *Helicobacter pylori* [42] and of MUC3 in inhibiting the attachment of pathogens such as *E. coli* in its secreted form [43]. The increase in mucin gene expression might be attributed to the ability of probiotic strains in either increasing the density of goblet cells or inducing heightened activity in already differentiated goblet cells [41]. Fermentation products of certain probiotic metabolites have also been reported to induce mucin expression [44]. Simultaneously, enhanced expression of MUC3 mucin gene might have barred the attachment of *Salmonella* to the intestinal epithelium, thereby preventing brain infection. Other co-incubation experiments with *L. plantarum* strain 299v and *Lactobacillus casei* GG have been observed to increase mucin secretion, resulting in reduced adherence and translocation of gut pathogens such as *E. coli* [45].

A significant decrease in the expression of BDNF gene was observed in mice suffering from *Salmonella* infection. BDNF has been reported to play an important role in neurogenesis, neurodegeneration and associated behavior [46, 47]. Various studies have reported its role in memory and learning owing to its ability in mediating plastic changes [48]. Additionally, dysregulation of this neurotrophic factor has been associated with psychiatric disorders such as depressive-like behaviour, bipolar disorder and schizophrenia [49–51]. Infection studies with *T. muris* as well as neonatal meningitis caused by *Streptococcus pneumoniae* have also reported similar findings which might be attributed to neurodegeneration caused by neuroinflammation [52]. However, normalization of BDNF expression was observed after administration of *L. plantarum* (RTA 8), thereby highlighting the ability of gut microbes in altering brain behaviour and neurochemistry which could be mediated via inflammation dependent as well as independent pathways.

## Conclusions

Given the complexity of the interactions and activation of multiple pathways during infection, the role of direct gut-brain communication holds enhanced significance. In our previous findings, mice in the *Salmonella* infected group exhibited depressive-like and anxiety-like behavioural changes [15]. Studies have reported the association of abnormal levels of serotonin, dopamine and norepinephrine, as well as the lower levels of BDNF, to various depressive and anxiety disorders [53], which might serve as another plausible reason for the pathophysiology observed therein. Studies using *C. jejuni* have also documented that local infection in the gut activates vagal sensory neurons via upregulation of c-Fos thereby leading to behavioural changes (anxiety and depressive like behaviour), in the absence of an overt immunological response [54]. In this context, the importance of these molecules in bi-directional communication, and the ability of microbes in influencing the same, have far reaching consequences in the field of gastrointestinal diseases and psychiatric disorders associated with depression and anxiety. It is here that the intervention of probiotics holds great potential. The crucial role of diet supplemented with such functional foods including probiotics and synbiotics, have been reported to provide health benefits that go beyond traditional nutrition thereby providing multi-dimensional and holistic health benefits to the host.

## Methods

### Bacterial strains

Two bacterial strains, namely *Salmonella enterica* serovar Typhimurium SL1344 and *Lactiplantibacillus plantarum* (RTA 8) were used throughout the study. The former was kindly gifted by Dr. Mrytyunjay Suar, Director of School of Biotechnology, KIIT University, Odisha and the latter was kindly provided by Prof. Rupinder Tewari, Department of Microbial Biotechnology, Panjab University, Chandigarh. *Salmonella* strain was routinely cultured in Luria–Bertani (LB) medium and streaked on solid LB agar plates containing antibiotic streptomycin at a concentration of 50 μg/ml. The *Lactiplantibacillus* strain was cultured in de Man, Rogosa and Sharpe (MRS) medium and maintained on MRS plates.

### Animals

Female BALB/c mice, 5–6 weeks old, weighing approx. 22–26 g, were used throughout the study. The animals were procured from Central Animal House, Panjab University, Chandigarh and adapted to the conditions of the animal room initially for a period of 1 week before initiation of any experiments. Animals were given standard pellet diet along with water ad libitum every day.

### In vivo study groups

Prior to initiation of experiments, mice were randomly divided into 3 groups containing 6–8 mice in each group.

#### Control group

All mice in this group served as control and each mouse was administered 0.1 ml of PBS.

#### Infected group

All mice in this group were individually infected with 10^8^ CFU/ml of *S. enterica* serovar Typhimurium SL1344 suspended in 0.1 ml of PBS.

#### Treatment group

All mice in this group were administered approx. 10^9^–10^10^ CFU/ml of *L. plantarum* (RTA 8) suspended in 0.2 ml of PBS daily for a period of 7 days before infecting them with 10^8^ CFU/ml of *S. enterica* serovar Typhimurium SL1344. Thereafter, mice in all the groups were sacrificed via cervical dislocation on day 7 post infection. Various vital organs of mice i.e. liver, spleen, intestine and brain were rapidly excised, weighted and stored at − 60 °C for preparation of homogenates. For *q*RT-PCR studies, the tissue sample was dissected at 0 °C and immediately transferred into vials containing RNA later solution and stored at − 80 °C until use.

### Evaluation of systemic *Salmonella* dissemination

Systemic spread of the pathogen from the gut to other organs and efficacy of the treatment with *L. plantarum* (RTA 8) was evaluated in terms of reduction in *Salmonella* bacterial count in vital organs such as the liver and the spleen as described previously [55]. Briefly, the liver and spleen tissues samples were rapidly excised and tissue homogenates (10%W/V) were prepared in ice cold PBS (0.05 M, pH 7.4). Appropriately diluted samples were then plated on streptomycin (50 μg/ml) containing LB agar plates with overnight incubation at 37 °C for bacterial count assessment.

### Parameters evaluated at the gut-brain axis

#### Intestinal and brain bio-burden

Dissemination of *Salmonella* from gut to brain along with the efficacy of *L. plantarum* (RTA 8) treatment was evaluated by enumeration of *Salmonella* bio-burden in intestinal and brain tissue homogenates as described above.

#### Level of neurotransmitters

Briefly, a part of the intestinal and brain tissue sample of mice in the above mentioned groups was homogenized in ice cold acidifying butanol solution using glass teflon homogenizer (50–75 mg tissue with 5 ml of HCl-butanol) for a period of 1 min. Thereafter, the homogenates were centrifuged at 2000 rpm for 20 min. The supernatant was aliquoted and 1 ml of this was then added to tube containing a mixture of 0.31 ml of 0.1 M HCl and 2.5 ml of heptane. After shaking vigorously for 10 min the tubes were then centrifuged again at 2000 rpm for 20 min. The aqueous phase (0.2 ml) was then used for assessment of neurotransmitters as mentioned below. All steps were carried out at 0 °C.

#### Estimation of dopamine

Level of dopamine in all the tissue homogenates was evaluated using the method as described by Carlsson and Waldeck [56]. Briefly, 0.5 ml 0.1 M PO_4_ buffer (pH 6.5) was added to 1 ml of above acid extract bringing the total volume to 3.8 ml by adding water. Thereafter, 0.05 ml of 0.02 N of iodine solution (consisting of 0.254 g iodine and 5 g KI in 5 ml water diluted to 100 ml) was added to the tubes. Then, after 5 min of incubation, 0.5 ml of alkaline sulphite solution was added to test sample but in case of blank, 0.5 ml of 5 N NaOH was added. After another 5 min, to all the samples, including the blank, 0.6 ml of 5 N acetic acid was added and all samples were then irradiated with UV (240 nm) for a duration of 10 min. This was followed by addition of 0.05 ml water to the test and 0.5 ml alkaline sulphite solution to blank. Thereafter, the fluorescence was measured at 335/410 nm. Values of unknown samples were obtained from the standard curve of dopamine (0.1–5 μg/ml).

#### Estimation of norepinephrine

Level of norepinephrine in all the tissue homogenates was evaluated using the method as described by Ciarlone [57]. Briefly, 0.1 ml of sodium acetate buffer (pH 6.9) and 0.05 ml of 0.4 M HCl was added to 0.2 ml of aqueous extract prepared as described above. This was followed by addition of 0.1 ml of iodine solution. After 2 min of incubation, 0.1 ml of alkaline sulphite solution was added to remove excess iodine. After each addition, all contents of the tube were mixed thoroughly. After 1.5 min, 0.1 ml of 10 N acetic acid was also added and mixed well. The tubes were then placed in a boiling water bath for a period of 6 min. The reading for NE was recorded at 395/485 nm after cooling the tubes under tap water. Values of the unknown samples were extrapolated from the standard curve of NE (0.1–5 μg/ml). In case of “blank”, the same procedure was followed except that thiosulphate reagent was added before addition of iodine.

#### Estimation of serotonin

Level of serotonin in all the tissue homogenates was evaluated as per the procedure given by Schlumpf et al. [58]. Briefly, 1.2 ml of ortho-phthalaldehyde (OPT) was added as to all the samples as well as blank (0.2 ml of 0.1 N hydrochloric acid) and mixed thoroughly. Thereafter, all tubes were placed in a boiling water bath for a period of 10 min. After cooling the samples under tap water, the fluorescence was read at 355/470 nm. Values of unknown samples were calculated from the standard plot of serotonin (0.1–5 μg/ml).

#### *q*RT-PCR studies

Total RNA from intestinal as well as brain tissues samples was extracted using TRIzol reagent (Ambion by Life Technologies). Complementary DNA (cDNA) was constructed using iScript cDNA Synthesis kit (Bio-Rad). Real-time PCR was performed using Applied Biosystem StepOne™ Real-Time PCR system with SYBR Green chemistry and SYBR Green Jumpstart Taq Ready Mix (Sigma-Aldrich) to amplify the cDNA template.

#### Evaluation of expression of mucin genes

Relative expression of two mucin genes, i.e., MUC 1 and MUC 3, were evaluated in all the groups by performing real time PCR studies. The primer sequences were as follows: MUC1 gene (Forward Primer: 5′-CAGTGCCAAGTCAATAC-3′, Reverse Primer: 5′-TGTTACTGGAGAAGGTAG-3′) and MUC3 gene (Forward Primer:5′-GTTGATGTCACCACTATG-3′, Reverse Primer: 5′-TGGTGTTGAGGTTAGAG-3′). They were designed using NCBI primer designing tool and synthesized by G Biosciences. For RT-PCR analysis, the following programme was employed: initial denaturation was performed at 94 °C for 2 min; which was followed by 40 cycles of denaturation at 94 °C for 15 s; annealing, extension and fluorescence was read at 55 °C for 1 min with 4 °C of optional hold.

#### Evaluation of expression of BDNF gene

Relative expression of BDNF gene was evaluated in all the groups by performing real time PCR studies. The primer sequences used were: BDNF gene (Forward Primer: 5′-CAAGAGTCCCGTCTGTACTTTAC-3′, Reverse Primer: 5′-GACTAGGGAAATGGGCTTAACA-3′). Primers were synthesized via IDT primer designing tool provided by IDT (Integrated DNA Technologies) and were synthesized from the same. For RT-PCR analysis, the following programme was employed: Initial denaturation was performed at 94 °C for 2 min; which was followed by 40 cycles of denaturation at 94 °C for 15 s; annealing, extension and fluorescence read at 60 °C for 1 min and 4 °C of optional hold.

For all the genes:

Melt curve analysis was performed by heating the samples from 55 to 95 °C with an increase of 0.5 °C and fluorescence was recorded. GAPDH was used as the housekeeping gene and the primer sequences were- Forward Primer: 5′-AACAGCAACTCCCACTCTTC-3′ and Reverse Primer: 5′-CCTGTTGCTGTAGCCGTATT-3′. Three biological replicates as well as three technical replicates were set up for each gene. Relative fold change in gene expression was assessed using the 2^−∆∆ct^ method.

#### Histological evaluation of gut and brain tissues

A part of the intestine as well as brain tissues from each group were stored in 10% buffered formalin. Briefly, the samples were then dehydrated using different grades of alcohol (70%, 80%, 90% and 100% absolute alcohol) after which they were washed in xylene for an hour and dipped in molten paraffin wax for crystallization and further sectioning. The sections were then kept in a water bath at 50 °C to remove wax and for mounting on a glass. Thereafter, the slides were further treated with xylene to remove wax and with ethanol to remove xylene. The slides were finally stained with hematoxylin, followed by eosin. Lastly, the slides were mounted in Distyrene Plasticizer Xylene (DPX) for histological investigation by examining under light microscope. The tissues were assessed for signs of inflammation and morphological changes.

#### Statistical analysis

All the values have been expressed as mean ± SD. Statistical analysis was performed using student’s *t *test, and two-way ANOVA which was followed by pairwise comparison using Tukey’s test. In all the tests*, p* ≤ 0.05 was considered as significant.

## Data Availability

All data supporting findings of the study has been included in the manuscript. Nucleotide sequence of *L. planatarum* (RTA 8) can be found in GenBank database under accession number: KJ802485. The NCBI mRNA reference sequence number for genes evaluated in the paper are as follows: Mus musculus mucin 1, transmembrane (Muc1): NM_013605.2, https://www.ncbi.nlm.nih.gov/nuccore/NM_013605.2. Mus musculus mucin 3, intestinal (Muc3): NM_010843.1, https://www.ncbi.nlm.nih.gov/nuccore/NM_010843.1. Mus musculus brain derived neurotrophic factor (Bdnf): NM_007540.4, https://www.ncbi.nlm.nih.gov/nuccore/NM_007540.4. Mus musculus glyceraldehyde-3-phosphate dehydrogenase (Gapdh): NM_001289726.1, https://www.ncbi.nlm.nih.gov/nuccore/NM_001289726.1.

## Abbreviations

ANS: Autonomic nervous system
BDNF: Brain-derived neurotrophic factor
cDNA: Complementary DNA
DA: Dopamine
EHEC: Enterohaemorrhagic *E. coli*
EMS: Emotional motor system
HPA: Hypothalamus–pituitary–adrenal axis
LPS: Lipopolysaccharide
MUC 1: Mucin 1 gene
MUC 3: Mucin 3 gene
OPT: Ortho-phthalaldehyde
SCFA: Short chain fatty acids
